# Advances in implementation strategies for treatment adherence in schizophrenia: a narrative review

**DOI:** 10.3389/fpsyt.2026.1752130

**Published:** 2026-02-09

**Authors:** Yan Shen, Xiaoli Wu

**Affiliations:** Department of Psychiatry, Lanxi Fifth Hospital, Lanxi, Zhejiang, China

**Keywords:** digital health, intervention, long-acting injectables, medication adherence, schizophrenia, treatment adherence

## Abstract

Schizophrenia is a chronic, relapsing mental illness where poor treatment adherence is a core challenge, significantly contributing to relapse and poor prognosis. Despite the efficacy of current pharmacotherapy, non-adherence rates are high. This review synthesizes the latest research on intervention strategies for improving treatment adherence in schizophrenia, exploring the effectiveness, limitations, and future directions of various approaches. We delve into behavioral and psychological interventions (including patient education, CBT, and motivational interviewing), pharmacological strategies (such as long-acting injectables), digital health technologies (like mobile apps, telemedicine, and wearable devices), and comprehensive, multidisciplinary care models. While each strategy demonstrates potential, they face challenges including a lack of standardized assessment tools, resource limitations, and the need for long-term efficacy data. Current research indicates a shift towards integrated and personalized interventions. We conclude that future efforts should focus on combining these diverse strategies into a holistic, patient-centered model, leveraging emerging technologies like AI and big data to provide more effective and sustainable support.

## 1 Introduction

Schizophrenia is a complex mental disorder affecting approximately 20 million people worldwide (1). Its core clinical features include positive symptoms (e.g., hallucinations, delusions), negative symptoms (e.g., blunted affect, avolition), and cognitive dysfunction (2, 3). The disease typically has its onset in adolescence or early adulthood and follows a protracted course, often accompanied by severe impairment in social and occupational functioning (4). Although the advent of antipsychotic medications has significantly improved the prognosis of schizophrenia, the relapse rate remains high; research indicates that the relapse rate within one year after treatment discontinuation can be as high as 80% (5). This not only causes significant distress to patients but also imposes a heavy economic burden on families and society (6). Treatment adherence in schizophrenia is a core issue affecting disease prognosis. Its high rates of non-adherence and complex influencing factors not only lead directly to high relapse rates and functional impairment but also increase healthcare costs significantly. Therefore, in-depth research and resolution of the adherence problem are of great clinical significance.

Treatment adherence generally refers to the consistency between a patient’s medication-taking behavior and the prescribed medical advice. Among patients with schizophrenia, poor adherence is a prevalent and long-standing unresolved problem. Epidemiological studies reveal an alarming situation: multiple large-scale surveys indicate that the treatment adherence rate among schizophrenia patients is only around 50% (7, 8), meaning that more than half of patients fail to take their medication as prescribed. This low adherence directly leads to a cascade of negative outcomes, significantly increasing the patient’s risk of relapse. Each relapse can exacerbate symptoms, further impair cognitive and social functioning, and lead to a progressive decline in academic, occupational, and interpersonal relationships (9). Long-term adherence problems also frequently lead to frequent hospitalizations and emergency department visits, imposing a significant burden on families and society. Furthermore, due to irregular medication intake, some patients exhibit “pseudo-treatment resistance” and are incorrectly diagnosed with treatment-resistant schizophrenia, thereby delaying appropriate treatment (10).

A deeper exploration of the causes of poor adherence reveals that its determinants are extremely complex, involving multiple levels such as the patient, the illness, medication, family, and the healthcare system. These factors are intertwined, posing a significant challenge for adherence interventions (11). At the patient level, barriers to medication-taking can include a lack of insight into the illness, impaired self-awareness, fear of medication side effects, and comorbidities (such as substance abuse) (12). Characteristics of the illness itself, such as severe negative symptoms and cognitive impairment, can also undermine a patient’s motivation and ability to adhere to medication (13). Meanwhile, the characteristics of the medication itself cannot be overlooked; the emergence of side effects (e.g., weight gain, extrapyramidal symptoms) is a significant reason for patients to independently reduce or discontinue their medication (14). The level of family support, family members’ understanding of the illness, as well as factors within the healthcare system, such as a strained patient-provider relationship and poor accessibility of medical services, also profoundly affect patient adherence (15). Therefore, no single intervention can comprehensively address the problem of adherence, prompting researchers to continuously explore multidimensional and comprehensive intervention strategies. In response to these complex influencing factors, this review will categorize and discuss the latest advances in different intervention strategies. These include behavioral and psychological interventions focused on changing patient cognition and behavior; pharmacological interventions aimed at simplifying medication regimens; digital health and telemedicine approaches utilizing emerging technologies; and comprehensive, multidisciplinary care models that integrate resources from multiple parties. The review will attempt to derive a comprehensive management plan to help improve treatment adherence in patients with schizophrenia.

## 2 Methodology

### 2.1 Search strategy

We conducted a comprehensive narrative review of the literature. A search of PubMed, PsycINFO, and Web of Science databases was performed for publications up to May 2025. Search terms were used in combination, including: (‘schizophrenia’ OR ‘psychosis’) AND (‘treatment adherence’ OR ‘medication adherence’ OR ‘compliance’ OR ‘persistence’) AND (‘intervention’ OR ‘strategy’ OR ‘management’ OR ‘long-acting injectable’ OR ‘digital health’ OR ‘telemedicine’ OR ‘CBT’ OR ‘psychoeducation’). Reference lists of key articles and systematic reviews were also manually screened for additional relevant publications.

### 2.2 Inclusion criteria

We included articles published in English that focused on intervention strategies to improve treatment adherence in patients with schizophrenia. Eligible articles included systematic reviews, meta-analyses, randomized controlled trials, observational studies, and narrative reviews that discussed behavioral, psychological, pharmacological, digital, or multidisciplinary interventions. We prioritized publications from the last 10 years but included older, foundational studies where relevant.

### 2.3 Study selection

Titles and abstracts of retrieved articles were screened for relevance by the authors. Full-text articles were then assessed for eligibility based on the inclusion criteria. Given the narrative nature of this review, strict systematic review criteria (such as PRISMA guidelines) were not fully applied. Instead, study selection was guided by relevance and methodological rigor. We prioritized ‘key studies’ defined as large-scale randomized controlled trials (RCTs), systematic reviews, and meta-analyses published in high-impact journals that have significantly influenced clinical guidelines or practice. While a formal quality appraisal tool (e.g., Cochrane Risk of Bias tool) was not utilized, we performed an implicit assessment of study quality. We prioritized studies with clear methodology, adequate sample sizes, and robust control conditions, while excluding small-scale pilot studies with inconclusive results unless they represented novel emerging technologies.

### 2.4 Data abstraction and charting

A formal data extraction form was not used, consistent with a narrative review methodology. Instead, information relevant to the review’s objectives was synthesized. We charted key information including the type of intervention (e.g., behavioral, pharmacological, digital), its mechanism, reported effectiveness, and identified limitations or challenges.

### 2.5 Data analysis and presentation

A narrative synthesis was employed to collate and summarize the findings. The results were organized thematically based on the different categories of intervention strategies (behavioral/psychological, pharmacological, digital health, and comprehensive models) to provide a comprehensive overview of the current landscape, challenges, and future directions.

### 2.6 Methodological justification and limitations

This review adopted a narrative approach rather than a systematic review methodology. This choice was justified by the broad scope of the topic, which encompasses highly heterogeneous intervention strategies—ranging from pharmacological (LAIs) to psychosocial (CBT, family therapy) and technological (digital health) approaches. A strict systematic meta-analysis would be difficult to conduct due to the variability in study designs, outcome measures, and patient populations across these diverse domains.

However, we acknowledge the inherent limitations of this approach. First, without a systematic search and strict inclusion/exclusion checklist for every available study, there is a potential for selection bias, where studies with positive results may be overrepresented. Second, the lack of a formal, standardized quality appraisal (risk of bias assessment) means that the findings presented here rely on the authors’ qualitative synthesis rather than a statistical aggregation of effect sizes. Finally, the interpretation of “high-impact” studies entails a degree of subjectivity. Readers should interpret the findings as a comprehensive, qualitative overview of the current landscape rather than a definitive quantitative evidence base.

## 3 Literature search

The initial database search yielded 1,240 articles. After removing duplicates, 890 titles and abstracts were screened. Of these, 210 full-text articles were assessed for eligibility. Finally, 62 key publications, including major clinical trials, systematic reviews, meta-analyses, and foundational reviews, were selected and included in this narrative synthesis. These studies formed the basis for analyzing the different categories of adherence interventions.

## 4 Advances in behavioral and psychological intervention strategies

Behavioral and psychological interventions are crucial non-pharmacological approaches to enhance adherence in patients with schizophrenia. They aim to fundamentally strengthen patients’ treatment motivation and self-management capabilities by modifying their cognition, behavioral patterns, and family support environments, thereby improving medication adherence and clinical outcomes.

## 5 Patient education and skills training

Patient education is the foundation for improving treatment adherence. Its core objective is to enhance the understanding of the illness and treatment for both patients and their families. The latest research emphasizes personalized and interactive educational methods. For example, using simple language, diagrams, and multimedia tools to explain illness knowledge, medication mechanisms of action, medication-taking techniques, and side-effect management strategies. Some studies have also introduced “skills training,” such as medication self-management training, which teaches patients how to set medication reminders, manage missed doses, and communicate with their doctors when side effects occur (16). Furthermore, work by Loots, McIntyre, and colleagues suggests that education alone is insufficient to guarantee behavioral change; successful interventions are typically a composite model combining “behavior” and “education.” Those interventions that integrate education with behavioral strategies (e.g., reminders, self-management training) are most effective in improving adherence (17, 18).

Although patient education and skills training are widely used as foundational interventions, existing research still has limitations. Most studies focus on short-term effectiveness, lacking follow-up on the long-term efficacy of the interventions. Additionally, the standardization of educational content and formats is low, leading to poor comparability between different study results. In recent findings by Al-Shashani et al., mobile applications (Apps) have shown great potential in providing standardized psychoeducation, medication reminders, and symptom monitoring (19). Moreover, a multi-center randomized controlled trial (RCT) in September 2025 pioneeringly tested a narrative-based digital psychoeducation, finding that this method, by enhancing emotional engagement and self-reflection, was significantly superior to traditional education in improving medication adherence and treatment attitudes, opening new avenues for the standardization of patient education (20). Future research needs to explore more personalized, quantifiable educational programs and focus on their long-term impact on patient adherence and quality of life.

## 6 Cognitive behavioral therapy

Cognitive Behavioral Therapy (CBT) is a psychotherapy that improves a patient’s psychological state by modifying their irrational cognitive and behavioral patterns (21). In adherence interventions, the primary application of CBT is to help patients identify and challenge erroneous beliefs about their illness and medication, such as “I don’t need to take medication” or “The medication is harmful to my body” (i.e., CBT for adherence, CBTa). Concurrently, by guiding patients to recognize the benefits of treatment, CBT can enhance their treatment motivation and help them set personal goals related to medication-taking, such as “Taking my medication on time allows me to work better.” A 2022 systematic review indicated that interventions combining CBT techniques with Motivational Interviewing (MI) show strong efficacy in improving and maintaining adherence behaviors, with effects remaining significant at 3 to 6-month follow-ups post-intervention (22). Furthermore, CBT also teaches patients skills to cope with medication-taking difficulties (e.g., forgetting medication, anxiety about side effects). A quasi-experimental study published in 2025 demonstrated that an 8-week CBT psychoeducation program not only significantly improved patient medication adherence (p=0.001) but also concurrently reduced their aggressive behaviors (23).

Despite these positive findings, the efficacy of CBT is not uniform across all patient profiles. Some evidence suggests that patients with severe cognitive impairment or profound negative symptoms (e.g., avolition) may struggle to engage with the cognitive restructuring tasks required in standard CBT (24). Furthermore, implementation in real-world settings faces systemic barriers. A systematic review of barriers to implementation highlighted that high caseloads, lack of protected time for clinicians, and insufficient funding often dilute the fidelity of CBT interventions compared to controlled trial settings (25). Therefore, adaptations such as shorter sessions or simplified content may be necessary for patients with lower cognitive functioning.

The effectiveness of CBT in improving adherence among schizophrenia patients has been confirmed by multiple studies, but its application in clinical practice still faces challenges. The main limitation is that implementing CBT requires professionally trained therapists, involves long treatment cycles, and is costly, which restricts its widespread adoption in large populations. Recent research by Lisa et al., addressing the time constraints of clinical settings (e.g., acute wards), explored the feasibility of brief, focused CBTp interventions (e.g., 6–8 sessions). Unfortunately, their results could only demonstrate that the brief CBTp intervention was effective, but they did not compare it with non-brief CBTp to obtain definitive conclusions (26). Furthermore, although work by Fulford et al. on digital CBT (dCBT) could improve the scalability of CBT (27), its effectiveness may be limited in patients with severe cognitive impairment or a profound lack of insight. Future research should explore how to integrate the core elements of CBT with more cost-effective intervention formats (such as digital therapeutics) to expand its beneficiary population.

## 7 Family intervention

The family is a crucial support system for patient recovery, and interventions aim to improve communication patterns among family members, reduce family burden, and enhance their support for the patient (6). Specific methods include imparting knowledge about schizophrenia, treatment plans, and the importance of adherence to family members (family psychoeducation), as well as resolving internal family conflicts, improving family functioning, and creating an environment conducive to the patient’s recovery and medication adherence (family therapy) (5). Multiple studies have shown that incorporating family intervention into multidisciplinary treatment programs can significantly improve patient adherence, reduce relapse, and enhance the quality of life for family members (5, 28, 29).

Although family intervention has made significant progress as an effective adherence support strategy, its research still has shortcomings. Existing studies are mostly small-sample studies, lacking large-scale, multi-center research to verify their generalizability. Moreover, family structures and functions vary greatly across different cultural backgrounds, and the cross-cultural adaptability of existing intervention models requires further study. The latest research progress has focused on two key areas: cultural adaptation and the simplification of intervention models. A 2021 systematic review on family interventions in non-Western cultural contexts emphasized the necessity of integrating interventions with local family structures, communication norms, and the degree of stigma associated with mental illness. For example, in Asian cultures where “High Expressed Emotion” (HEE) may manifest as overprotection rather than criticism, the focus of intervention should shift towards improving boundaries and promoting patient agency (30). Additionally, to overcome barriers to large-scale implementation (e.g., high cost, long duration), brief family intervention models are becoming a focal point (Brief Family Psychoeducation). Work by Katsuki et al. suggests that a brief family psychoeducation (BFPE) model, consisting of only 6 sessions, is as effective as traditional long-term (9-month) family therapy in improving patient adherence and reducing 12-month relapse rates, but with significantly higher cost-effectiveness. This provides a feasible solution for resource-limited settings. In the future, research needs to develop more culturally sensitive family intervention models and evaluate their effectiveness in different social and cultural environments.

However, the concept of ‘expressed emotion’ (EE) itself varies across cultures. In many collectivist societies (e.g., parts of Asia, Latin America, and Africa), behaviors labeled as ‘emotional over-involvement’ in the West are often perceived as normative ‘caring’ rather than pathological intrusive behaviors (31). Therefore, interventions must be culturally adapted to harness this family cohesion rather than pathologize it. For instance, the ‘Culturally-adapted Family Intervention’ (CaFI) model specifically tailored for African-Caribbean families emphasizes shared understanding and spiritual coping mechanisms, demonstrating that culturally aligned protocols can significantly improve engagement and retention rates compared to standard protocols (31). Future research should prioritize developing ‘culturally congruent’ family therapies that respect local hierarchies and communication styles.

## 8 Motivational interviewing

Motivational Interviewing (MI) is a patient-centered, directive communication style aimed at eliciting and enhancing a patient’s intrinsic motivation to change (32). In adherence interventions, its core principles are: expressing empathy by listening to the patient’s concerns and ambivalence; developing discrepancy by helping the patient see the discrepancies between the negative consequences of non-adherence and the positive outcomes of adherence; rolling with resistance by not directly confronting the patient’s resistance but rather guiding them toward self-discovery; and supporting self-efficacy by reinforcing their confidence in their ability to successfully take medication (32).

As a non-confrontational intervention technique, MI shows good promise in improving treatment attitudes and adherence in patients with schizophrenia (33). However, the effectiveness of this technique is highly dependent on the practitioner’s communication skills and clinical experience, thus posing challenges for its implementation in clinical settings. Existing research mostly focuses on evaluating short-term intervention effects; further research is needed on the long-term impact of MI on adherence. In this regard, research by Chien et al. has yielded mixed results: the short-term (within 3 months) improvement in adherence with MI is clear, but the maintenance of its medium- to long-term (6–12 months) effects remains uncertain, suggesting that regular “booster sessions” may be necessary to maintain motivation (34). It is also important to note that findings regarding MI’s effectiveness on adherence are mixed. While some studies report significant improvements, others fail to demonstrate a clear advantage over standard care, potentially due to variations in therapist fidelity and the complexity of the patient-therapist relationship in schizophrenia (35). This inconsistency suggests that MI may be most effective when used as a specific engagement strategy for ‘pre-contemplative’ patients rather than a standalone maintenance treatment. Furthermore, how to effectively integrate MI with other interventions (such as medication education) to achieve synergistic effects is also an important future research direction. To address the limitation of MI’s dependency on practitioner skill, an important trend is to combine it with structured therapies. A 2023 study exploring combined Motivational Interviewing-Cognitive Behavioral Therapy (MI-CBT) found that using MI as a “lead-in” intervention significantly increased patient engagement in the subsequent CBT, thereby indirectly reinforcing adherence (36) (**Table 1**).

**Table 1:** Specific methods and strategies of motivational interviewing in schizophrenia adherence research.

Research Type	Interview Method	Interview Target	Effectiveness Evaluation Indicators	Involved Research Literature
Randomized Controlled Trial (RCT)	Structured Motivational Interviewing, combined with medication education	Schizophrenia patients	Adherence score, relapse rate, length of hospital stay	(34)
Cohort Study	Motivational Interviewing vs. standard treatment control	Schizophrenia patients	Adherence, symptom severity, quality of life	(37, 38)
Meta-analysis	Systematic review and meta-analysis, summarizing multiple studies	Schizophrenia patients	Adherence improvement effect (effect size)	(11)
Qualitative Research	In-depth interviews, open-ended questionnaires	Patients, family members, medical staff / healthcare providers	Patients' views on the interview experience, adherence barriers	(39, 40)

## 9 Pharmacological interventions: recent applications of long-acting injectables

Pharmacological interventions, particularly the application of novel Long-Acting Injectables (LAIs), effectively address the challenge of poor adherence to oral medications by fundamentally changing the route of administration, thereby providing patients with schizophrenia a more reliable and stable treatment option.

## 10 Mechanism, advantages, and limitations of LAIs

Long-Acting Injectables (LAIs) are antipsychotic medications formulated as sustained-release preparations, administered via intramuscular injection, which allows for slow release within the body, thereby maintaining stable plasma concentrations for several weeks or even months (41). This delivery method fundamentally bypasses the problem of patient adherence to daily oral medication, ensuring the continuity of treatment. Its primary advantages are that it eliminates the need for daily dosing, reducing adherence problems caused by forgetfulness or medication refusal, while also avoiding the plasma concentration fluctuations seen with variable dosing intervals of oral medications (42). Furthermore, if a patient fails to receive an injection on time, clinicians and family members can immediately detect it, enabling early intervention. It also alleviates the patient’s psychological burden associated with daily medication, thereby improving their quality of life (43).

In recent years, several novel LAIs have become available, offering more clinical choices. For example, Paliperidone Palmitate is available in 1-month and 3-month formulations, and studies have shown it to be significantly effective in relapse prevention with good tolerability (44, 45). Aripiprazole LAI is available in 1-month and 2-month formulations, characterized by a narrower side-effect profile, particularly a lower risk of extrapyramidal symptoms and weight gain (41, 46, 47). A large-scale real-world study comparing the effectiveness of different LAIs found that their use was associated with significantly lower hospitalization rates and improved quality of life compared to oral medications (48).

Despite the significant advantages of LAIs in improving adherence and clinical outcomes, their application still faces several limitations. First, the injection itself may cause anxiety and discomfort, and some patients may refuse treatment due to a fear of injections. Second, the cost of LAIs is typically higher than that of oral medications, posing a significant barrier in regions with limited healthcare resources. Furthermore, if adverse drug reactions occur, they are difficult to resolve quickly due to the long duration of action of the medication, necessitating prolonged clinical management. Finally, the dosing intervals for LAIs are typically fixed, lacking the flexibility for adjustment afforded by oral medications. This can be inconvenient during the initial treatment phase when frequent dose titration may be necessary. Beyond physical discomfort, ethical concerns regarding patient autonomy constitute a significant barrier. The use of LAIs can sometimes be perceived by patients as ‘chemical restraint’ or a coercive measure, potentially damaging the therapeutic alliance if not introduced collaboratively (49). Clinicians may inadvertently contribute to this stigma by reserving LAIs as a ‘last resort’ for non-compliant patients rather than presenting them as a convenient first-line option. Addressing these perceptions through shared decision-making frameworks is crucial to improve the acceptability of LAIs (**Table 2**).

**Table 2:** Overview of common long-acting injectable (LAI) antipsychotics for schizophrenia.

Drug Name	Route of Administration	Dosing Frequency	Primary Recommendation Level	Key Study / Reference
Fluphenazine	Intramuscular (IM)	2-4 weeks	First-generation, recommended	(50)
Haloperidol	Intramuscular (IM)	2-4 weeks	First-generation, recommended	(50)
Risperidone	Intramuscular (IM)	2 weeks	Recommended by multiple guidelines	(51)
Paliperidone	Intramuscular (IM)	1 month or 3 months	Recommended by multiple guidelines, especially as first-line therapy	(52)
Aripiprazole	Intramuscular (IM)	1 month or 2 months	Recommended by multiple guidelines	(53)
Ziprasidone	Intramuscular (IM)	2 weeks	Recommended by some guidelines	(54)
Olanzapine	Intramuscular (IM)	2-4 weeks	Recommended by some guidelines	(55)

## 11 Development status and bottlenecks of novel LAIs

The current research and development of novel LAIs are primarily focused on extending dosing intervals, reducing injection volumes, and expanding the range of drug types. Researchers are actively exploring novel sustained-release technologies, such as microspheres, nanocrystal suspensions, and implantable drug delivery systems, aiming to achieve longer drug release durations. This endeavor seeks to develop LAIs that require injection only semi-annually or even annually, thereby further simplifying treatment regimens and enhancing patient adherence (56). Concurrently, another critical direction in R&D is the development of LAIs with fewer metabolic side effects to improve patients’ quality of life. Copolymer-based delivery technologies (e.g., MedinCell’s BEPO technology) have been successfully applied. A recent example is the 1-month and 2-month subcutaneous injection formulation of Risperidone (TV-46000/Uzedy). This technology allows the drug to form a biodegradable polymer matrix subcutaneously, from which the drug is slowly released. Its advantages include rapidly reaching therapeutic concentrations post-administration (without the need for oral supplementation) and a small injection volume (57).

However, the R&D of LAIs still faces numerous bottlenecks. First, the technical challenges are significant, requiring assurance that the drug can be released in the body at a precise and constant rate while balancing safety and efficacy. Second, clinical trial cycles are long and costly, necessitating extended follow-up to evaluate long-term effects and adverse reactions, which increases the financial burden of development. Furthermore, how to convert effective medications that do not yet have LAI formulations (such as clozapine) into an injectable form without compromising their efficacy and safety remains an unresolved challenge. In the future, these bottlenecks are expected to be gradually overcome with continuous breakthroughs in drug delivery technology and a deeper understanding of drug metabolism mechanisms.

## 12 Application of digital health and telemedicine in adherence management

The rise of digital health technologies and telemedicine has provided entirely new and innovative solutions for adherence management in schizophrenia. Through mobile applications, remote consultations, and wearable devices, they enable real-time monitoring and intervention in patients’ treatment behaviors, enhancing the accessibility and convenience of care.

With the popularization of smartphones, mobile applications have become an emerging tool for adherence management. Their functions include reminding patients to take medication via alarms and push notifications; helping patients and clinicians monitor illness fluctuations in real-time through mood and symptom tracking to adjust treatment plans promptly; providing health education on illness knowledge, healthy diet, and exercise guidance; and motivating patients to adhere to medication schedules through gamification mechanisms such as points and badges. Multiple randomized controlled trials have shown that mobile applications integrating various functions can significantly improve medication adherence in patients with schizophrenia, especially in the short term. A systematic review and meta-analysis focusing on severe mental illness (SMI) confirmed this, finding that digital technology interventions (especially those based on smartphone APPs) effectively improve medication adherence (58).

Meanwhile, telemedicine developed rapidly during the pandemic. Through remote video consultations, patients can maintain communication with their doctors and ensure treatment continuity, even if they are unable to visit the hospital due to geographical distance or mobility issues. Remote consultation allows patients to communicate with clinicians in a familiar environment, alleviating their anxiety. Meta-analyses have shown that telemedicine interventions are comparable to traditional face-to-face consultations in adherence management and are associated with high patient satisfaction (59).

Furthermore, emerging wearable devices and biosensors also offer possibilities for objectively assessing adherence. For instance, smart pillboxes can record the time a patient accesses medication, while ingestible sensors (digital pills) transmit a signal after being swallowed, monitoring medication intake in real-time (60). Such technologies can provide precise adherence data, helping clinicians more accurately judge a patient’s medication status and thus deliver more targeted interventions, holding immense promise for the future.

## 13 Current research directions

Current research in digital health for adherence management is primarily focused on developing more personalized intervention programs. For example, utilizing artificial intelligence (AI) and machine learning (ML) algorithms to analyze big data on patients’ medication-taking behaviors and lifestyles to predict adherence risks and provide precise, personalized intervention strategies. Recent studies indicate that machine learning models can integrate data from electronic health records (EHRs), smartphone sensors, and patient-reported outcomes to predict future (e.g., within 30 days) medication non-adherence events show promise in preliminary studies (AUC > 0.80), thereby enabling “Just-in-Time Adaptive Interventions” (JITAI) (61). However, caution is warranted, as many of these models lack external validation across diverse patient populations, raising concerns about algorithmic bias and their generalizability to real-world clinical settings (62). Additionally, researchers are actively exploring the integration of virtual reality (VR) and augmented reality (AR) technologies into psychoeducation and skills training, aiming to create immersive, interactive therapeutic experiences to enhance patient engagement and treatment motivation. Another important direction is the development of platforms that can seamlessly integrate multiple intervention modalities, such as combining mobile apps, wearable devices, and telemedicine services into a single digital ecosystem, to achieve comprehensive, real-time monitoring and management of patient adherence (63).

## 14 Research limitations

Despite the vast promise of digital health technologies in adherence management, their application still faces multiple limitations. First, many studies lack long-term, large-scale randomized controlled trials to validate their sustained effectiveness in real-world settings. Second, the efficacy of digital health tools is highly dependent on patient acceptance and technological literacy; adherence and usage rates may be lower among elderly patients or those with severe cognitive impairments. This ‘digital divide’ is particularly pronounced in older adults with schizophrenia and those from lower socioeconomic backgrounds who may lack reliable internet access or the latest smartphones. A recent study on digital exclusion emphasized that without targeted digital literacy training, the deployment of sophisticated e-health tools might inadvertently widen health disparities, leaving the most vulnerable populations behind (64). And, user engagement remains a critical challenge, often described as ‘digital attrition.’ While initial uptake of mental health apps may be high, real-world data suggest that long-term retention is often low. A systematic analysis of mental health apps revealed that the median retention rate at 30 days was only 3.3%, indicating a significant gap between installation and sustained therapeutic use (65). Furthermore, data privacy and security are critical challenges in digital health applications, necessitating strict regulations and technical safeguards to protect sensitive patient medical information. Ethical concerns regarding the ‘black box’ nature of AI algorithms and the potential for depersonalization of care must also be addressed before widespread adoption (66). Moreover, a ‘cultural mismatch’ in digital design remains a largely unaddressed barrier. Most commercially available mental health apps are developed in Western, high-income countries (WEIRD societies), often failing to accommodate linguistic diversity, differing health literacy levels, or culturally specific explanatory models of mental illness (67). For example, direct translation of CBT content without cultural adaptation (e.g., modifying metaphors or examples to fit local contexts) has been shown to reduce user engagement and therapeutic alliance in non-Western populations (67). Addressing these disparities requires a shift from ‘one-size-fits-all’ global apps to locally co-designed digital solutions. Finally, the development costs of digital health tools are high, and their generalizability across different cultural and healthcare systems requires further research and validation (68).

## 15 Implementation strategies for comprehensive, multidisciplinary care models

The comprehensive, multidisciplinary care model emphasizes a patient-centered approach, aiming to provide patients with more holistic and systematic adherence management for better long-term outcomes. It achieves this by building a comprehensive, continuous treatment support system through the integration of resources from psychiatry, primary care, community rehabilitation, and social support networks.

The core of this model is collaborative care, which breaks down the barriers between psychiatric and physical medical care. A multidisciplinary team (including psychiatrists, nurses, social workers, etc.) collaboratively provides services to the patient. Recent research has demonstrated that this model can significantly improve adherence and clinical outcomes for patients with schizophrenia, particularly for those with physical comorbidities (69). A Cochrane review of “Assertive Community Treatment” (ACT) showed that, compared to standard care, the ACT model significantly reduced patient hospitalization duration and was more effective at keeping patients engaged with services (i.e., adherence in a broader sense). Furthermore, this model extends treatment from the hospital into the community. It enhances patients’ social functioning through community rehabilitation activities (e.g., vocational rehabilitation, social skills training) and integrates medication management, psychological counseling, rehabilitation training, and social support through comprehensive, one-stop clinics. The social support network also plays a vital role in patient recovery and adherence maintenance. For example, community workers can conduct regular home visits and provide medication reminders, while peer supporters—patients who have recovered well—can share their experiences, providing role models and emotional support for new patients. This form of support is particularly effective in enhancing patient treatment motivation (70).

## 16 Research directions and developmental bottlenecks

Currently, research on comprehensive, multidisciplinary care models is moving towards multi-dimensional integration and personalization. Future studies will focus on exploring how to seamlessly integrate different intervention strategies (e.g., pharmacotherapy, psychological counseling, and community support) into a unified care framework to achieve synergistic effects. Additionally, research is focusing on how to utilize data analytics and machine learning techniques to create personalized care pathways for different patient populations, thereby maximizing adherence improvement.

However, the promotion and application of this model still face numerous challenges. The first is implementation complexity; it requires coordinating resources from different medical and community organizations, incurring high inter-departmental communication and management costs. The second is resource limitation; a lack of professionally trained multidisciplinary teams, especially at the primary care and community levels, severely restricts the model’s generalizability. Finally, there are research bottlenecks. Existing studies are mostly small-scale pilot projects, lacking large-scale, long-term, multi-center randomized controlled trials to comprehensively evaluate long-term efficacy and cost-effectiveness. It is also difficult to determine which specific intervention combinations are most effective for particular patient groups. Future efforts must overcome these bottlenecks by developing standardized implementation guidelines, strengthening inter-agency collaboration, and utilizing technological means to optimize resource allocation.

## 17 Integrative framework: a stage-based approach

**Figure 1:**
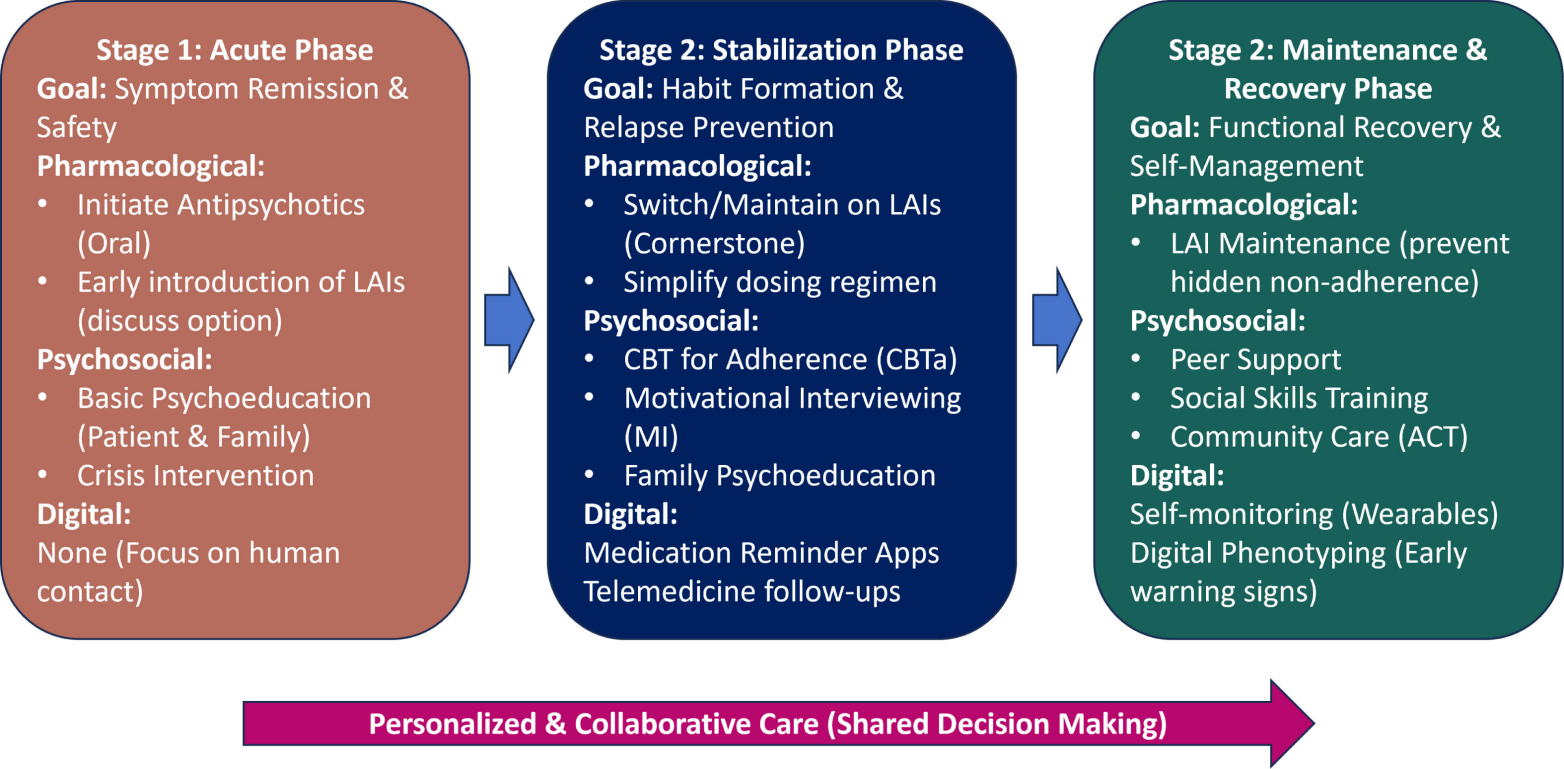
An integrated, stage-based intervention framework for treatment adherence in schizophrenia. The model categorizes interventions into three clinical phases, Acute, Stabilization, and Maintenance, highlighting the synergistic use of pharmacotherapy (especially LAIs), psychosocial support (CBT, MI), and digital health tools.

To synthesize the diverse strategies discussed in this review and address the complexity of adherence management, we propose an integrative, stage-based conceptual framework (**Figure 1**). This model illustrates how pharmacological, psychosocial, and digital interventions can be synergistically combined across different phases of the illness.

In the Acute Phase, the primary goal is symptom control; interventions focus on establishing a therapeutic alliance, early introduction of LAIs to ensure medication delivery, and basic family psychoeducation. As the patient moves to the Stabilization Phase, the focus shifts to habit formation. Here, LAIs serve as the pharmacological cornerstone, augmented by CBT for adherence (CBTa) to address cognitive barriers and mobile apps for daily reminders. Finally, in the Maintenance Phase, the objective is functional recovery. Interventions expand to include peer support, community-based rehabilitation (e.g., ACT), and advanced digital monitoring (e.g., wearables) to detect early warning signs of relapse. Throughout all stages, a personalized, patient-centered approach rooted in shared decision-making remains the foundation.

## 18 Conclusion and future perspectives

Despite significant progress in adherence intervention research, multiple challenges and limitations remain. First, at the methodological level, the lack of standardized, reliable adherence assessment tools leads to poor comparability between study results. Simultaneously, the heterogeneity of study samples (e.g., differences in disease type, illness duration, cultural background) also limits the generalizability of findings. Furthermore, most studies focus on short-term effectiveness, lacking in-depth data on the long-term efficacy of interventions.

Looking ahead, adherence interventions will evolve towards personalization, utilizing genomics and biomarkers to predict patient adherence, thereby enabling the formulation of precise intervention plans at the outset of treatment. Meanwhile, the application of artificial intelligence and big data will allow us to more accurately analyze patient behavior patterns and medication data, thereby predicting adherence risks and providing timely interventions. The future trend is to organically integrate the various intervention strategies mentioned above into an efficient, comprehensive solution. For example, using LAIs as the cornerstone of adherence, supplemented by digital health tools for daily reminders and tracking, and combined with family and community support, will form a comprehensive, multi-dimensional adherence management model. This multi-dimensional, personalized, and integrated intervention approach will provide more effective and sustainable treatment support for patients with schizophrenia.

Treatment adherence in schizophrenia is a critical factor influencing patient prognosis and quality of life. This review has systematically summarized the latest research progress in various intervention strategies, including behavioral and psychological interventions, long-acting injectables, digital health, and comprehensive care models. Despite significant achievements, challenges remain in standardized assessment, long-term efficacy, and personalized intervention. Future research should focus on developing multidisciplinary, personalized, and integrated intervention protocols, combined with emerging technologies, to provide more effective and sustainable treatment support for patients with schizophrenia.

## References

[1] Same K Shobeiri P Rashidi M-M Ghasemi E Saeedi Moghaddam S Mohammadi E . A global, regional, and national burden and quality of care index for schizophrenia: global burden of disease systematic analysis 1990-2019. Schizophr Bull. (2024) 50:1083–93. doi: 10.1093/schbul/sbad120, PMID: 37738499 PMC11349008

[2] Faden J Citrome L . Schizophrenia: one name, many different manifestations. Med Clinics North America. (2023) 107:61–72. doi: 10.1016/j.mcna.2022.05.005, PMID: 36402500

[3] Marder SR Umbricht D . Negative symptoms in schizophrenia: Newly emerging measurements, pathways, and treatments. Schizophr Res. (2023) 258:71–7. doi: 10.1016/j.schres.2023.07.010, PMID: 37517366

[4] Correll CU Arango C Fagerlund B Galderisi S Kas MJ Leucht S . Identification and treatment of individuals with childhood-onset and early-onset schizophrenia. Eur Neuropsychopharmacol. (2024) 82:57–71. doi: 10.1016/j.euroneuro.2024.02.005, PMID: 38492329

[5] Bighelli I Rodolico A García-Mieres H Pitschel-Walz G Hansen W-P Schneider-Thoma J . Psychosocial and psychological interventions for relapse prevention in schizophrenia: a systematic review and network meta-analysis. Lancet Psychiatry. (2021) 8:969–80. doi: 10.1016/S2215-0366(21)00243-1, PMID: 34653393

[6] Rodolico A Bighelli I Avanzato C Concerto C Cutrufelli P Mineo L . Family interventions for relapse prevention in schizophrenia: a systematic review and network meta-analysis. Lancet Psychiatry. (2022) 9:211–21. doi: 10.1016/S2215-0366(21)00437-5, PMID: 35093198

[7] Acosta FJ Hernández JL Pereira J Herrera J Rodríguez CJ . Medication adherence in schizophrenia. World J Psychiatry. (2012) 2:74–82. doi: 10.5498/wjp.v2.i5.74, PMID: 24175171 PMC3782179

[8] Nyanyiwa S Peters K Murphy G . A scoping review: Treatment attitudes and adherence for adults with schizophrenia. J Clin Nurs. (2022) 31:3060–75. doi: 10.1111/jocn.16219, PMID: 35043496

[9] Arango C Fagiolini A Gorwood P Kane JM Diaz-Mendoza S Sahota N . Delphi panel to obtain clinical consensus about using long-acting injectable antipsychotics to treat first-episode and early-phase schizophrenia: treatment goals and approaches to functional recovery. BMC Psychiatry. (2023) 23:453. doi: 10.1186/s12888-023-04928-0, PMID: 37344763 PMC10286361

[10] Emsley R Chiliza B Asmal L . The evidence for illness progression after relapse in schizophrenia. Schizophr Res. (2013) 148:117–21. doi: 10.1016/j.schres.2013.05.016, PMID: 23756298

[11] Li IH Hsieh WL Liu WI . A systematic review and meta-analysis of the effectiveness of adherence therapy and its treatment duration in patients with schizophrenia spectrum disorders. PPA. (2023) 17:769–80. doi: 10.2147/PPA.S401650, PMID: 36974078 PMC10039634

[12] Rose B Harvey PD . Anosognosia in schizophrenia. CNS Spectr. (2025) 30:e24. doi: 10.1017/S1092852924002323, PMID: 39721947 PMC13064738

[13] Selvan P Devkare P Shetty A Dharmadhikari S Khandhedia C Mane A . A review on the pharmacology of cariprazine and its role in the treatment of negative symptoms of schizophrenia. Front Psychiatry. (2024) 15:1385925. doi: 10.3389/fpsyt.2024.1385925, PMID: 38711874 PMC11071166

[14] Leucht S Priller J Davis JM . Antipsychotic drugs: A concise review of history, classification, indications, mechanism, efficacy, side effects, dosing, and clinical application. AJP. (2024) 181:865–78. doi: 10.1176/appi.ajp.20240738, PMID: 39350614

[15] Semahegn A Torpey K Manu A Assefa N Tesfaye G Ankomah A . Psychotropic medication non-adherence and its associated factors among patients with major psychiatric disorders: a systematic review and meta-analysis. Syst Rev. (2020) 9:17. doi: 10.1186/s13643-020-1274-3, PMID: 31948489 PMC6966860

[16] Arsenault-Mehta K Hochman-Bérard M Johnson A Semenova D Nguyen B Willis J . Pharmacological management of neurocognitive impairment in schizophrenia: A narrative review. Neuropsychopharm Rep. (2024) 44:2–16. doi: 10.1002/npr2.12382, PMID: 37794723 PMC10932777

[17] Loots E Goossens E Vanwesemael T Morrens M Van Rompaey B Dilles T . Interventions to improve medication adherence in patients with schizophrenia or bipolar disorders: A systematic review and meta-analysis. IJERPH. (2021) 18:10213. doi: 10.3390/ijerph181910213, PMID: 34639510 PMC8508496

[18] McIntyre RS Berk M Brietzke E Goldstein BI López-Jaramillo C Kessing LV . Bipolar disorders. Lancet. (2020) 396:1841–56. doi: 10.1016/S0140-6736(20)31544-0, PMID: 33278937

[19] Al-Shashani A Abu Sabra MA Al-Gamal E . The impact of using digital health interventions and psychoeducation on medication adherence among patients with schizophrenia: A scoping review. Issues Ment Health Nurs. (2025) 46:735–45. doi: 10.1080/01612840.2025.2492694, PMID: 40300193

[20] Liu Z Zhu D Zhang Z Liu Y Yang H Chang F . Effects of a narrative-based psychoeducational intervention on medication adherence in individuals with schizophrenia: a multicentre, parallel-group randomised controlled trial. eClinicalMedicine. (2025) 88:103483. doi: 10.1016/j.eclinm.2025.103483, PMID: 40979217 PMC12445229

[21] Guaiana G Abbatecola M Aali G Tarantino F Ebuenyi ID Lucarini V . Cognitive behavioural therapy (group) for schizophrenia. Cochrane Database System Rev. (2022) 2022:1. doi: 10.1002/14651858.CD009608.pub2, PMID: 35866377 PMC9308944

[22] Inwanna S Duangchan C Matthews AK . Effectiveness of interventions to promote medication adherence in schizophrenic populations in Thailand: A systematic review. IJERPH. (2022) 19:2887. doi: 10.3390/ijerph19052887, PMID: 35270585 PMC8910437

[23] Can SY Budak FK . The effect of cognitive behavioural therapy–based psychoeducation on medication adherence and aggression in individuals diagnosed with schizophrenia: an experimental study. Psychiatr Ment Health Nurs. (2025) 32:445–56. doi: 10.1111/jpm.13127, PMID: 39445584 PMC11891425

[24] Jauhar S McKenna PJ Radua J Fung E Salvador R Laws KR . Cognitive-behavioural therapy for the symptoms of schizophrenia: systematic review and meta-analysis with examination of potential bias. Br J Psychiatry. (2014) 204:20–9. doi: 10.1192/bjp.bp.112.116285, PMID: 24385461

[25] Kopelovich SL Stiles B Monroe-DeVita M Hardy K Hallgren K Turkington D . Psychosis REACH: effects of a brief CBT-informed training for family and caregivers of individuals with psychosis. Psychiatr Serv. (2021) 72:1254–60. doi: 10.1176/appi.ps.202000740, PMID: 34015942

[26] Wood L Butterworth H Nyikavaranda P Ariyo A Malde-Shah N Guerin E . Patient’s experiences of a cognitive behaviour therapy informed crisis intervention for psychosis delivered in inpatient settings: A qualitative exploration. Clin Psychol Psychoth. (2024) 31:e3033. doi: 10.1002/cpp.3033, PMID: 39089290

[27] Fulford D Correll CU Harvey PD Cohen AS . Digital therapeutics for people with schizophrenia spectrum disorders: A systematic literature review of their effect on symptoms and functioning. Schizophr Bull. (2025), sbaf134. doi: 10.1093/schbul/sbaf134, PMID: 40854230 PMC13391626

[28] Solmi M Croatto G Piva G Rosson S Fusar-Poli P Rubio JM . Efficacy and acceptability of psychosocial interventions in schizophrenia: systematic overview and quality appraisal of the meta-analytic evidence. Mol Psychiatry. (2023) 28:354–68. doi: 10.1038/s41380-022-01727-z, PMID: 35999275

[29] Valery K-M Prouteau A . Schizophrenia stigma in mental health professionals and associated factors: A systematic review. Psychiatry Res. (2020) 290:113068. doi: 10.1016/j.psychres.2020.113068, PMID: 32474069

[30] Husain MO Khoso AB Renwick L Kiran T Saeed S Lane S . Culturally adapted family intervention for schizophrenia in Pakistan: a feasibility study. Int J Psychiatry Clin Pract. (2021) 25:258–67. doi: 10.1080/13651501.2020.1819332, PMID: 32930011

[31] Degnan A Baker S Edge D Nottidge W Noke M Press CJ . The nature and efficacy of culturally-adapted psychosocial interventions for schizophrenia: a systematic review and meta-analysis. Psychol Med. (2018) 48:714–27. doi: 10.1017/S0033291717002264, PMID: 28830574

[32] Lu EY Cheng ASK Tsang HWH Chen J Leung S Yip A . Psychoeducation, motivational interviewing, cognitive remediation training, and/or social skills training in combination for psychosocial functioning of patients with schizophrenia spectrum disorders: A systematic review and meta-analysis of randomized controlled trials. Front Psychiatry. (2022) 13:899840. doi: 10.3389/fpsyt.2022.899840, PMID: 36245879 PMC9561245

[33] Wang W Chau AKC Kong P Sun X So SH . Efficacy of motivational interviewing in treating co-occurring psychosis and substance use disorder: A systematic review and meta-analysis. J Clin Psychiatry. (2021) 83:1. doi: 10.4088/JCP.21r13916, PMID: 34963202

[34] Chien WT Mui JH Cheung EF Gray R . Effects of motivational interviewing-based adherence therapy for schizophrenia spectrum disorders: a randomized controlled trial. Trials. (2015) 16:270. doi: 10.1186/s13063-015-0785-z, PMID: 26072311 PMC4469254

[35] Dobber J Latour C van Meijel B Ter Riet G Barkhof E Peters R . Active ingredients and mechanisms of change in motivational interviewing for medication adherence. A mixed methods study of patient-therapist interaction in patients with schizophrenia. Front Psychiatry. (2020) 11:78. doi: 10.3389/fpsyt.2020.00078, PMID: 32265746 PMC7105777

[36] Reddy LF Glynn SM McGovern JE Sugar CA Reavis EA Green MF . A novel psychosocial intervention for motivational negative symptoms in schizophrenia: combined motivational interviewing and CBT. AJP. (2023) 180:367–76. doi: 10.1176/appi.ajp.20220243, PMID: 36891649

[37] Drake RJ Nordentoft M Haddock G Arango C Fleischhacker WW Glenthøj B . Modeling determinants of medication attitudes and poor adherence in early nonaffective psychosis: implications for intervention. Schizophr Bull. (2015) 41:584–96. doi: 10.1093/schbul/sbv015, PMID: 25750247 PMC4393703

[38] Zygmunt A Olfson M Boyer CA Mechanic D . Interventions to improve medication adherence in schizophrenia. AJP. (2002) 159:1653–64. doi: 10.1176/appi.ajp.159.10.1653, PMID: 12359668

[39] Gyllensten AL Jacobsen LN Gard G . Clinician perspectives of Basic Body Awareness Therapy (BBAT) in mental health physical therapy: An international qualitative study. J Bodywork Mov Ther. (2019) 23:746–51. doi: 10.1016/j.jbmt.2019.04.012, PMID: 31733757

[40] Tsuck-Ram N Moka A Lavi-Rotenberg A Igra L Hasson-Ohayon I . Subjective experience and perceived benefits in clients with schizophrenia following participation in metacognition reflection and insight therapy (MERIT). Behav Sci. (2024) 14:450. doi: 10.3390/bs14060450, PMID: 38920781 PMC11200425

[41] Correll CU Kim E Sliwa JK Hamm W Gopal S Mathews M . Pharmacokinetic characteristics of long-acting injectable antipsychotics for schizophrenia: an overview. CNS Drugs. (2021) 35:39–59. doi: 10.1007/s40263-020-00779-5, PMID: 33507525 PMC7873121

[42] Kishimoto T Hagi K Kurokawa S Kane JM Correll CU . Long-acting injectable versus oral antipsychotics for the maintenance treatment of schizophrenia: a systematic review and comparative meta-analysis of randomised, cohort, and pre–post studies. Lancet Psychiatry. (2021) 8:387–404. doi: 10.1016/S2215-0366(21)00039-0, PMID: 33862018

[43] Wang D Schneider-Thoma J Siafis S Qin M Wu H Zhu Y . Efficacy, acceptability and side-effects of oral versus long-acting- injectables antipsychotics: Systematic review and network meta-analysis. Eur Neuropsychopharmacol. (2024) 83:11–8. doi: 10.1016/j.euroneuro.2024.03.003, PMID: 38490016

[44] Valsecchi P Barlati S Garozzo A Deste G Nibbio G Turrina C . Paliperidone palmitate in short- and long-term treatment of schizophrenia. Rivista di Psichiatr. (2019) 54:235–248. doi: 10.1708/3281.32542, PMID: 31909750

[45] Najarian D Sanga P Wang S Lim P Singh A Robertson MJ . A randomized, double-blind, multicenter, noninferiority study comparing paliperidone palmitate 6-month versus the 3-month long-acting injectable in patients with schizophrenia. Int J Neuropsychopharmacol. (2022) 25:238–51. doi: 10.1093/ijnp/pyab071, PMID: 34791283 PMC8929757

[46] Preda A Shapiro BB . A safety evaluation of aripiprazole in the treatment of schizophrenia. Expert Opin Drug Saf. (2020) 19:1529–38. doi: 10.1080/14740338.2020.1832990, PMID: 33064050

[47] Rudå D Jensen KG Decara MS Klauber DG Fagerlund B Møllegaard JR . CYP2D6 genotyping and antipsychotic-associated extrapyramidal adverse effects in a randomized trial of aripiprazole versus quetiapine extended release in children and adolescents, aged 12–17 years, with first episode psychosis. J Clin Psychopharmacol. (2021) 41:667–72. doi: 10.1097/JCP.0000000000001490, PMID: 34735099

[48] Lin D Thompson-Leduc P Ghelerter I Nguyen H Lafeuille M-H Benson C . Real-world evidence of the clinical and economic impact of long-acting injectable versus oral antipsychotics among patients with schizophrenia in the United States: A systematic review and meta-analysis. CNS Drugs. (2021) 35:469–81. doi: 10.1007/s40263-021-00815-y, PMID: 33909272 PMC8144083

[49] Roberts LW Geppert CMA . Ethical use of long-acting medications in the treatment of severe and persistent mental illnesses. Compr Psychiatry. (2004) 45:161–7. doi: 10.1016/j.comppsych.2004.02.003, PMID: 15124145

[50] Chouinard G Annable L Campbell W . A randomized clinical trial of haloperidol decanoate and fluphenazine decanoate in the outpatient treatment of schizophrenia. J Clin Psychopharmacol. (1989) 9:247–53. doi: 10.1097/00004714-198908000-00003, PMID: 2570086

[51] Lasser RA Bossie CA Zhu Y Locklear JC Kane JM . Long-acting risperidone in young adults with early schizophrenia or schizoaffective illness. Ann Clin Psychiatry. (2007) 19:65–71. doi: 10.1080/10401230701332931, PMID: 17612845

[52] Li H Turkoz I Zhang F . Efficacy and safety of once-monthly injection of paliperidone palmitate in hospitalized Asian patients with acute exacerbated schizophrenia: an open-label, prospective, noncomparative study. Neuropsychiatr Dis Treat. (2016) 12:15–24. doi: 10.2147/NDT.S83651, PMID: 26730193 PMC4694691

[53] Kane JM Sanchez R Perry PP Jin N Johnson BR Forbes RA . Aripiprazole intramuscular depot as maintenance treatment in patients with schizophrenia: a 52-week, multicenter, randomized, double-blind, placebo-controlled study. J Clin Psychiatry. (2012) 73:617–24. doi: 10.4088/JCP.11m07530, PMID: 22697189

[54] Leucht S Crippa A Siafis S Patel MX Orsini N Davis JM . Dose-response meta-analysis of antipsychotic drugs for acute schizophrenia. Am J Psychiatry. (2020) 177:342–53. doi: 10.1176/appi.ajp.2019.19010034, PMID: 31838873

[55] Kane JM Detke HC Naber D Sethuraman G Lin DY Bergstrom RF . Olanzapine long-acting injection: a 24-week, randomized, double-blind trial of maintenance treatment in patients with schizophrenia. Am J Psychiatry. (2010) 167:181–9. doi: 10.1176/appi.ajp.2009.07081221, PMID: 20008947

[56] Alidori S Subramanian R Holm R . Patient-centric long-acting injectable and implantable platforms─An industrial perspective. Mol Pharm. (2024) 21:4238–58. doi: 10.1021/acs.molpharmaceut.4c00665, PMID: 39160132 PMC11372838

[57] Kane JM Harary E Eshet R Tohami O Weiser M Leucht S . Efficacy and safety of TV-46000, a long-acting, subcutaneous, injectable formulation of risperidone, for schizophrenia: a randomised clinical trial in the USA and Bulgaria. Lancet Psychiatry. (2023) 10:934–43. doi: 10.1016/S2215-0366(23)00288-2, PMID: 37924833

[58] Richey AG Kovacs I Browne S . Use of an ingestible, sensor-based digital adherence system to strengthen the therapeutic relationship in serious mental illness. JMIR Ment Health. (2022) 9:e39047. doi: 10.2196/39047, PMID: 36459392 PMC9758639

[59] Cowan T Cohen AS Raugh IM Strauss GP . Ambulatory audio and video recording for digital phenotyping in schizophrenia: Adherence & data usability. Psychiatry Res. (2022) 311:114485. doi: 10.1016/j.psychres.2022.114485, PMID: 35276573 PMC9018573

[60] Taub S Krivoy A Whiskey E Shergill SS . New approaches to antipsychotic medication adherence - safety, tolerability and acceptability. Expert Opin Drug Saf. (2022) 21:517–24. doi: 10.1080/14740338.2021.1983540, PMID: 34541978

[61] Ekpezu AO Wiafe I Oinas-Kukkonen H . Predicting adherence to behavior change support systems using machine learning: systematic review. JMIR AI. (2023) 2:e46779. doi: 10.2196/46779, PMID: 38875538 PMC11041458

[62] Torous J Bucci S Bell IH Kessing LV Faurholt-Jepsen M Whelan P . The growing field of digital psychiatry: current evidence and the future of apps, social media, chatbots, and virtual reality. World Psychiatry. (2021) 20:318–35. doi: 10.1002/wps.20883, PMID: 34505369 PMC8429349

[63] Lane E D’Arcey J Kidd S Onyeaka H Alon N Joshi D . Digital phenotyping in adults with schizophrenia: A narrative review. Curr Psychiatry Rep. (2023) 25:699–706. doi: 10.1007/s11920-023-01467-z, PMID: 37861979

[64] Greer B Robotham D Simblett S Curtis H Griffiths H Wykes T . Digital exclusion among mental health service users: qualitative investigation. J Med Internet Res. (2019) 21:e11696. doi: 10.2196/11696, PMID: 30626564 PMC6329420

[65] Baumel A Muench F Edan S Kane JM . Objective user engagement with mental health apps: systematic search and panel-based usage analysis. J Med Internet Res. (2019) 21:e14567. doi: 10.2196/14567, PMID: 31573916 PMC6785720

[66] Fiske A Henningsen P Buyx A . Your robot therapist will see you now: ethical implications of embodied artificial intelligence in psychiatry, psychology, and psychotherapy. J Med Internet Res. (2019) 21:e13216. doi: 10.2196/13216, PMID: 31094356 PMC6532335

[67] Spanhel K Balci S Feldhahn F Bengel J Baumeister H Sander LB . Cultural adaptation of internet- and mobile-based interventions for mental disorders: a systematic review. NPJ Digit Med. (2021) 4:128. doi: 10.1038/s41746-021-00498-1, PMID: 34433875 PMC8387403

[68] Liem A Natari RB Jimmy null Hall BJ . Digital health applications in mental health care for immigrants and refugees: A rapid review. Telemed J E Health. (2021) 27:3–16. doi: 10.1089/tmj.2020.0012, PMID: 32498658

[69] Kisely SR Campbell LA . Compulsory community and involuntary outpatient treatment for people with severe mental disorders. Schizophr Bull. (2015) 41:542–3. doi: 10.1093/schbul/sbv021, PMID: 25767194 PMC4393705

[70] Lloyd-Evans B Mayo-Wilson E Harrison B Istead H Brown E Pilling S . A systematic review and meta-analysis of randomised controlled trials of peer support for people with severe mental illness. BMC Psychiatry. (2014) 14:39. doi: 10.1186/1471-244X-14-39, PMID: 24528545 PMC3933205

